# Secreted Phospholipases A_2_ in Hereditary Angioedema With C1-Inhibitor Deficiency

**DOI:** 10.3389/fimmu.2018.01721

**Published:** 2018-07-23

**Authors:** Stefania Loffredo, Anne Lise Ferrara, Maria Bova, Francesco Borriello, Chiara Suffritti, Nóra Veszeli, Angelica Petraroli, Maria Rosaria Galdiero, Gilda Varricchi, Francescopaolo Granata, Andrea Zanichelli, Henriette Farkas, Marco Cicardi, Gérard Lambeau, Gianni Marone

**Affiliations:** ^1^Department of Translational Medical Sciences, Center for Basic and Clinical Immunology Research (CISI), University of Naples Federico II, WAO Center of Excellence, Naples, Italy; ^2^Division of Gastroenterology, Boston Children’s Hospital, Harvard Medical School, Boston, MA, United States; ^3^Department of Biomedical and Clinical Sciences, University of Milan, Luigi Sacco Hospital Milan, Milan, Italy; ^4^Hungarian Angioedema Center, 3rd Department of Internal Medicine, Semmelweis University, Budapest, Hungary; ^5^Université Côte d’Azur, CNRS, Institut de Pharmacologie Moléculaire et Cellulaire, Valbonne Sophia Antipolis, France; ^6^Institute of Experimental Endocrinology and Oncology “G. Salvatore”, National Research Council, Naples, Italy

**Keywords:** hereditary angioedema, angiogenesis, angiopoietins, C1 inhibitor deficiency, vascular endothelial growth factor, vascular permeability, phospholipase A_2_

## Abstract

**Background:**

Hereditary angioedema (HAE) caused by deficiency (type I) or dysfunction (type II) of the C1 inhibitor protein (C1-INH-HAE) is a disabling, potentially fatal condition characterized by recurrent episodes of swelling. We have recently found that patients with C1-INH-HAE have increased plasma levels of vascular endothelial growth factors and angiopoietins (Angs), which have been associated with vascular permeability in several diseases. Among these and other factors, blood endothelial cells and vascular permeability can be modulated by extracellular or secreted phospholipases A_2_ (sPLA_2_s).

**Objective:**

We sought to investigate the enzymatic activity and biological functions of sPLA_2_ in patients with C1-INH-HAE.

**Methods:**

sPLA_2_s enzymatic activity was evaluated in the plasma from 109 adult patients with C1-INH-HAE and 68 healthy donors in symptom-free period and attacks. Plasma level of group IIA sPLA_2_ (hGIIA) protein was measured in selected samples. The effect of C1-INH-HAE plasma on endothelial permeability was examined *in vitro* using a vascular permeability assay. The role of hGIIA was determined using highly specific sPLA_2_ indole inhibitors. The effect of recombinant hGIIA on C1-INH activity was examined *in vitro* by functional assay.

**Results:**

Plasma sPLA_2_ activity and hGIIA levels are increased in symptom-free C1-INH-HAE patients compared with controls. sPLA_2_ activity negatively correlates with C1-INH protein level and function. C1-INH-HAE plasma increases endothelial permeability *in vitro*, and this effect is partially reverted by a specific hGIIA enzymatic inhibitor. Finally, recombinant hGIIA inhibits C1-INH activity *in vitro*.

**Conclusion:**

sPLA_2_ enzymatic activity (likely attributable to hGIIA), which is increased in C1-INH-HAE patients, can promote vascular permeability and impairs C1-INH activity. Our results may pave the way for investigating the functions of sPLA_2_s (in particular, hGIIA) in the pathophysiology of C1-INH-HAE and may inform the development of new therapeutic targets.

## Introduction

Hereditary angioedema due to C1-inhibitor deficiency (C1-INH-HAE) is a disabling, potentially fatal condition characterized by recurrent episodes of swelling caused by reduced levels (type I) or dysfunction (type II) of the C1-INH protein (1, 2). These patients display insufficient C1-INH function to prevent bradykinin (BK) formation (3), which increases endothelial permeability and leads to recurrent episodes of swelling (i.e., angioedema attacks) involving the deeper layers of the skin and/or submucosal tissue (4–6). High concentrations of circulating BK and cleaved high-molecular weight kininogen (HK) are elevated in patients with C1-INH-HAE and further increased during angioedema attacks, with the latter correlating with attack frequency (7, 8). In addition, specific BK antagonism reverts angioedema symptoms (9). Even if, BK stands as main mediator of C1-INH-HAE (10, 11), the need to explain symptom variability among patients genetically deficient in C1-INH prompt on identifying additional factors that modulate endothelial cell biology and vascular permeability (12).

Vascular endothelial growth factors (VEGFs) and angiopoietins (Angs) have well-established role in endothelial cells conditioning and modulation of permeability (13–16) and we recently showed their increase in plasma of patients with C1-INH-HAE in symptom-free period (17). Interestingly, variants in angiopoietins 1 gene (ANGPT1) are associated with a recently described form of hereditary angioedema (HAE) (18). Nevertheless, other factors controlling endothelial cell biology and vascular permeability may intervene in C1-INH-HAE.

Phospholipase A_2_ (PLA_2_) enzymes hydrolyze the fatty acid from membrane glycerophospholipids releasing arachidonic acid, and lysophospholipid (19, 20). The superfamily of PLA_2_ comprises different proteins that can be divided into six classes (20, 21). Secreted or extracellular PLA_2_s (sPLA_2_s) directly modulates endothelial cell migration and vascular permeability *in vitro*. The effects of sPLA_2_s depend on their enzymatic activity and ability to engage different targets [e.g., PLA_2_R1, heparan sulfate proteoglycans (HSPGs), integrins] (22–31). They play critical roles in several pathophysiological processes. Indeed, sPLA_2_s activate several immune cell subsets (25, 30–33) and are expressed in inflamed tissues and tumors (19, 20, 34, 35).

Owing to the ability of sPLA_2_s to modulate vascular permeability (either by directly activating endothelial cells or by catalyzing the production/degradation of vasoactive molecules) (36), we have analyzed the enzymatic activity and biological function of sPLA_2_s present in plasma from C1-INH-HAE patients in symptom-free period and during attacks.

## Materials and Methods

### Reagents

The following were purchased: bovine aortic endothelial cells (BAEC) (Thermo Fisher Scientific^©^, San Jose, CA, USA); bovine serum albumin, l-glutamine, antibiotic–antimycotic solution (10,000 IU/ml penicillin, 10 mg/ml streptomycin, and 25 µg/ml amphotericin B), Heparinase I and III Blend from *Flavobacterium heparinum*, DMEM and fetal calf serum (endotoxin level <0.1 EU/ml) (MP Biomedicals Europe, Illkirch, France). Antibody anti-VEGF-A, anti-Ang1, and anti-Ang2 (R&D System, Minneapolis, MN, USA). The recombinant human secreted phospholipase A_2_ group IIA (hGIIA) was prepared as described (37) and the inhibitor Me-Indoxam (38) and RO032107A (39) were obtained from Dr. Michael Gelb (University of Washington, Seattle, WA, USA). All other reagents were from Carlo Erba (Milan, Italy).

### Study Population

We studied 109 C1-INH-HAE patients followed at the University of Milan, University of Naples Federico II, and University of Budapest and 68 normal healthy controls. Diagnosis of C1-INH-HAE was based on the presence of at least one clinical and laboratory criteria as described (40). Table 1 summarizes the clinical characteristics of patients with C1-INH-HAE and healthy controls. Patients (104 type I and 5 type II C1-INH-HAE) belong to 76 unrelated families and their median age at symptoms onset was 6 years (interquartile range 3–14). There were no differences between the study groups in terms of age, sex, and ethnicity. The frequency of angioedema attacks was used as an index of disease severity; therefore, patients were grouped according to the number of attacks registered during the last 12 months: 69 of them had less than 12 attacks (low frequency), while 40 complained ≥12 attacks (high frequency). Regarding symptom-free samples, blood sampling was performed at least 8 days apart from an angioedema attack in all patients. In 22 patients, blood samples were obtained also during angioedema attack. Nineteen patients were taking prophylactic therapy at the time of blood collection (4 were on tranexamic acid and 15 on attenuated androgens).

**Table 1:** Characteristics of 109 patients with C1-INH-HAE and 68 healthy donor controls.

Characteristics	Healthy donors (*n* = 68)	Patients (*n* = 109)
Age—years^a^	32 (28–40)	37 (23–48)
Gender male—no. (%)	32 (47%)	38 (34.9%)
Caucasian race—%	100%	100%
Unrelated families	NA	76
Age at onset—years^a^	NA	6 (3–14)
C1-INH-HAE—no. (%)		
Type I	NA	104 (95.4%)
Type II	NA	5 (4.5%)
Prophylaxis—no. (%)		
Tranexamic acid	NA	4 (3.8%)
Attenuated androgens	NA	15 (10%)
≥12 attacks/year—no. (%)	NA	40 (36.7%)

*^a^Data are expressed as median values (interquartile ranges). Data were analyzed by *t-*test*.

*NA, not applicable*.

### Blood Sampling

The Ethical Committee of the University of Naples Federico II, University of Milan, and University of Budapest approved that plasma obtained during routine diagnostics could be used for research investigating the physiopathology of HAE and written informed consent was obtained from patients according to the principles expressed in the Declaration of Helsinki. Blood was collected during routine diagnostic procedures and the remaining plasma sample was labeled with a code which was documented into a datasheet. The controls had been referred for routine medical check-up and volunteered for the study by giving informed consent. Technicians who performed the assays were blinded to the patients’ history. Blood was drawn by a clean venepuncture and minimal stasis using two types of anticoagulants: sodium citrate 3.2% and, for the measurement of cleaved HK, an inhibitor cocktail containing 100 mM benzamidine, 400 µg/ml hexadimethrine bromide, 2 mg/ml soybean trypsin inhibitor, 263 µM leupeptin, and 20 mM aminoethylbenzene-sulphonyl fluoride dissolved in acid/citrate/dextrose (100 mM trisodium citrate, 67 mM citric acid, and 2% dextrose, pH 4.5). After centrifugation (2,000 *g* for 20 min at 22°), the plasma was divided into aliquots and stored at −80°C until used.

### Complement System Analysis

Tube with an anti-coagulant sodium citrate 3.2% is used for separating plasma from whole blood. Plasma C1-INH was measured by radial immunodiffusion (NOR-Partigen, Siemens Healthcare Diagnostics, Munich, Germany). C4 antigen levels in Italia was measured by radial immunodiffusion (NOR-Partigen) (the method is not specific for C4 fragments) whereas in Hungary C4 levels was measured by turbidimetry (Roche Cobas Integra 800, Beckman Coulter Complement C4). The antibody employed in the Beckman Coulter C4 assay is directed against the common portion of the C4 molecule and it exhibit the same reactivity with C4 fragments as well as with the native molecule.

C1-INH function was assessed as the capacity of plasma to inhibit the esterase activity of exogenous C1s as measured on a specific chromogenic substrate by means of a commercially available kit (Technoclone GmbH, Vienna, Austria) (17). Reference ranges were 0.70–1.30 U C1-INH/ml (1 U C1-INH corresponds to the average C1-INH activity present in 1 ml of fresh citrated normal plasma). The functional activity of C1-INH was also expressed as a percentage of activity of C1-INH present in samples. Normal values of activity of C1-INH are greater than 0.7 U C1 INH/ml (>70%). According to diagnostic criteria, all patients enrolled in this study had C1-INH functional activity lower than 50% of normal (41). In selected experiments, plasma of healthy controls was incubated (2 h, 37°C) with and without hGIIA (0.5–5 µg/ml). After treatment, enzymatic activity of C1-inhibitor was assessed using commercially available MicroVue C1-Inhibitor EIA kit (Quidel, San Diego, CA, USA).

### Determination of VEGFs and Angs

Plasma levels of angiogenic and lymphangiogenic mediators were measured using commercially available ELISA kits for VEGF-A, VEGF-C, Ang1, and Ang2 (R&D System, Minneapolis, MN, USA) according to the manufacturer’s instructions (17). The ELISA sensitivity is 31.1–2,000 pg/ml for VEGF-A, 62–4,000 pg/ml for VEGF-C, 156.25–10,000 pg/ml for Ang1, and 31.1–4,000 pg/ml for Ang2.

### Contact System Analysis

The cleavage of HK was assessed by means of sodium dodecyl sulfate-polyacrylamide gel electrophoresis (SDS-PAGE) and immunoblotting analysis (a modification of the method described by Berrettini et al.) (42). Samples were loaded on a 9% SDS-PAGE. After electrophoretic separation, proteins were transferred from the gel to a polyvinylidene difluoride membrane using Bio-Rad Trans-Blot^®^ Turbo™ Transfer System (Bio-Rad Laboratories, Hercules, CA, USA). HK was identified using goat polyclonal anti-HK light chain (Nordic, Tilburg, The Netherlands) and visualized using a biotinylated rabbit anti-goat antibody (Sigma Aldrich Co., St. Louis, MO, USA). The density of the bands obtained was measured using a Bio-Rad GS-800 densitometer. The amount of cleaved HK was expressed as a percentage of total HK.

### PLA_2_ Activity Assay

Activity of PLA_2_ in plasma of patients and healthy controls was measured by Life Technologies EnzChek^®^phospholipase A_2_ assay. Briefly, a PLA_2_ substrate cocktail consisting of 7-hydroxycoumarinyl-arachidonate (0.3 mM), 7-hydroxycoumarinyl-linolenate (0.3 mM), hydroxycoumarinyl 6-heptenoate (0.3 mM), dioleoylphosphatidylcholine (DOPC) (10 mM), and dioleoylphosphatidylglycerol (DOPG) (10 mM) was prepared in ethanol. Liposomes were formed by gradually adding 77 µl substrate/lipid cocktail to 10 ml of PLA_2_ buffer (50 mM Tris–HCl, 100 mM NaCl, 1 mM CaCl_2_) while stirring rapidly over 1 min using a magnetic stirrer Fluorescence (excitation at 360 nm and emission at 460 nm) was measured and specific activity [relative fluorescent units (RFU)/ml] for each sample was calculated. Plasma (50 µl) of patients and healthy controls was added to 96-well plates, and PLA_2_ activity was evaluated by adding 50 µl of substrate cocktail. In selected experiments, plasma of patients with C1-INH-HAE was incubated (20 min, 37°C) with Me-Indoxam (100 nM) and RO032107A (100 nM) or control medium. At the end of incubation, PLA_2_ activity was measured.

### ELISA for hGIIA

Human sPLA_2_ group IIA levels in plasma samples were determined by ELISA kit (Catalog No. MBS9303777, MyBioSource, San Diego, CA, USA). The concentration of hGIIA in plasma was tested in duplicate and determined against a standard curve for each ELISA assay. To evaluate the reliability of the assay we loaded 500 and 1,000 pg/ml of recombinant hGIIA in the ELISA wells and the spectrophotometer measured 397 and 886 pg/ml, respectively.

### *In Vitro* Vascular Permeability Assay

Endothelial cell permeability was assessed by *in vitro* vascular permeability assay kit (Life Technologies, Carlsbad, CA, USA). BEAC were seeded onto collagen-coated Transwell filters (1 µm pore size) at the density of 7.5 × 10^4^ cells/well in a 96-well receiver plate and incubated at 37°C and 5% CO_2_ for 72 h. After this time, cells starvation step was performed by adding DMEM 0.5% FBS and incubation for 18 h at 37°C, 5% CO_2_. Plasma from patients with C1-INH-HAE was pre-incubated (20 min, 37°C) with Me-Indoxam (100 nM), RO032107A (100 nM), anti-VEGF-A (1 µg/ml), anti-Ang2 (1 µg/ml), anti-Ang1 (1 µg/ml), or control medium. BAEC were then pre-incubated (30 min, 37°C) with heparinase (0.4 U/ml) or control medium and then stimulated (18 h, 37°C) with plasma of healthy controls, or with plasma of C1-INH-HAE patients or with the combination of C1-INH-HAE plasma with Me-Indoxam, RO032107A, anti-VEGF-A, anti-Ang2, and anti-Ang1. To evaluate vascular permeability, a high-molecular weight FITC-Dextran was added on top of the cells, allowing the fluorescent molecules to pass through the endothelial cell monolayer at a rate proportional to the monolayer’s permeability. The extent of permeability was determined by measuring the fluorescence (485 nm excitation and 535 nm emission) using Sunrise™ spectrofluorometer (Tecan) and RFU was calculated.

### Statistical Analysis

Data were analyzed with the GraphPad Prism 5 software package. Data were tested for normality using the D’Agostino–Pearson normality test. If normality was not rejected at 0.05 significance level, we used parametric tests. Otherwise, for not-normally distributed data we used nonparametric tests. Statistical analysis was performed by unpaired two-tailed *t*-test or two-tailed Mann–Whitney test as indicated in figure legends. Correlations between two variables were assessed by Spearman’s correlation analysis and reported as coefficient of correlation (*r*). Plasma activity of sPLA_2_ and hGIIA is shown as the median (horizontal black line), the 25th and 75th percentiles (boxes) and the 5th and 95th percentiles (whiskers) of 68 controls and 109 patients. In selected experiments, the data are expressed as mean values ± SD of the indicated number of experiments. Statistical analysis was performed with Prism 5 (GraphPad Software) by one-way analysis of variance followed by Dunnett’s test (when comparison was made against a control). Statistically significant differences were accepted when the *p* value was ≤0.05.

## Results

### Increased Plasma Levels of sPLA_2_ Enzymatic Activity and hGIIA Protein in Patients with C1-INH-HAE compared with Healthy Controls

**Figure 1:**
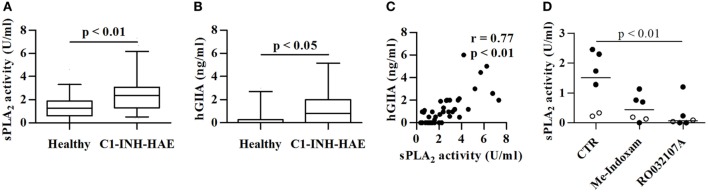
Plasma levels sPLA_2_ activity and hGIIA protein in symptom-free with C1-INH-HAE patients and healthy controls. Data are shown as the median (horizontal black line), the 25th and 75th percentiles (boxes) and the 5th and 95th percentiles (whiskers) of 68 healthy controls and 109 C1-INH-HAE patients for sPLA_2_ **(A)** and 36 controls and patients for hGIIA assessment **(B)**. Correlation between sPLA_2_ and hGIIA **(C)** was assessed by Spearman’s correlation analysis and reported as coefficient of correlation (*r*). **(D)** Plasma of patients with C1-INH-HAE (black circles) and of healthy controls (white circles) was pre-incubated (20 min, 37°C) with Me-Indoxam (100 nM), RO032107A (100 nM), or control medium. At the end of incubation, sPLA_2_ activity was evaluated.

We assessed the PLA_2_ enzymatic activity (likely attributable to sPLA_2_) in the plasma from 109 C1-INH-HAE patients in the symptom-free period vs 68 healthy controls matched for age and gender (Table 1). Figure 1A shows that sPLA_2_ activity was increased by appreciatively twofold in C1-INH-HAE patients in symptom-free period compared with controls [sPLA_2_: 2.4 (1.3–3.0) vs 1.3 (0.6–1.8) U/ml median values (interquartile ranges)]. No gender or age differences in sPLA_2_ activity were found in both controls and patients and (see Table S1 and Figure S1 in Supplementary Material).

Several sPLA_2_s have been identified in mammals (IB, IIA, IIC, IID, IIE, IIF, III, V, X, XIIA, XIIB, and otoconin-95) (27), with hGIIA being the most represented in human serum and plasma (43–47). Accordingly, we found increased levels of hGIIA protein in C1-INH-HAE patients compared with controls (Figure 1B). The sPLA_2_ enzymatic activity in plasma from C1-INH-HAE patients strongly correlated with hGIIA plasma levels (*r* = 0.77; *p* < 0.01) (Figure 1C). The enzymatic activity was significantly inhibited by Me-Indoxam (an enzymatic inhibitor of several sPLA_2_s) (38) and RO032107A (a specific hGIIA inhibitor) (39) in both C1-INH-HAE patients and healthy donors (Figure 1D). Collectively, these results indicate that the increased sPLA_2_ enzymatic activity observed in the plasma from C1-INH-HAE patients is likely attributable to higher levels of hGIIA protein.

### The sPLA_2_ Activity in Plasma inversely correlates with C1-INH Functional Activity and Protein Levels

**Figure 2:**
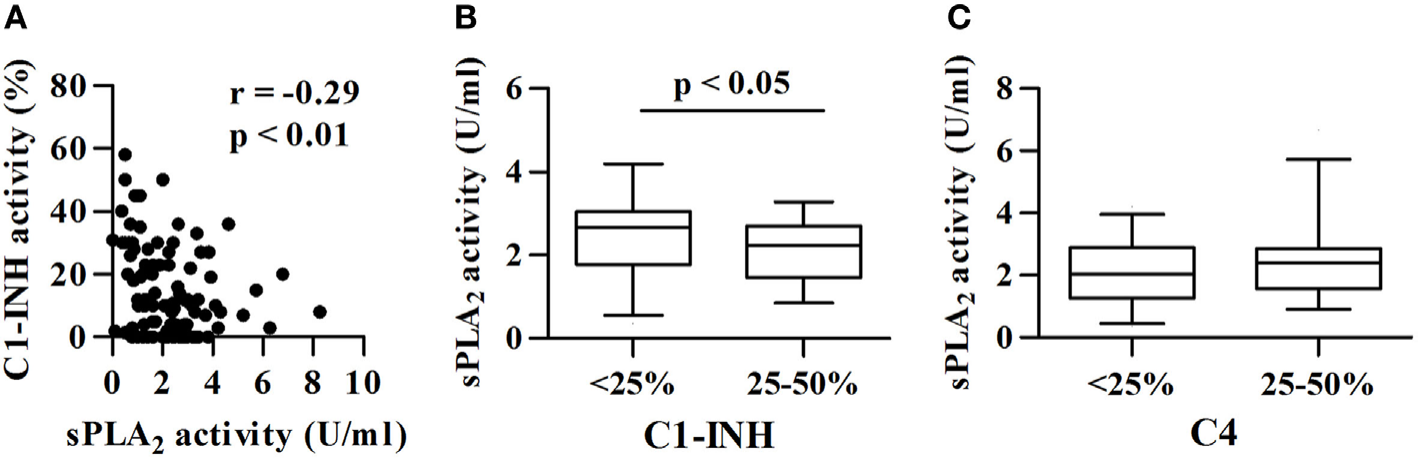
Correlations between sPLA_2_ activity and complement components. sPLA_2_ activity in patients with different functional activity of C1-INH. **(A)** Correlation between sPLA_2_ and C1-INH activity was assessed by Spearman’s correlation analysis and reported as coefficient of correlation (*r*). **(B)** Patients were divided in two groups: C1-INH type I patients with less than 25 or 25–50% of normal C1-INH protein values. **(C)** Patients were divided in two groups: C1-INH-HAE patients with less than 25 or 25–50% of normal C4 values. Activity of sPLA_2_ **(B,C)** is shown.

In C1-INH-HAE patients, functional C1-INH levels are, by definition, below 50% of the normal value (40) and C4 concentrations are usually reduced and can be used as a screening test (48). We investigated whether differences in the complement component levels (C1-INH and C4) were associated with differences in sPLA_2_ activity. C1-INH activity negatively correlated with sPLA_2_ activity (*r* = −0.29; *p* < 0.01) (Figure 2A). Moreover, C1-INH-HAE type I patients with lower C1-INH protein concentration (less than 25% of normal values) had higher plasma activity of sPLA_2_s than patients with higher C1-INH protein concentration (25–50% of normal values) [2.6 (1.8–3.0) vs 2.2 (1.4–2.7) U/ml] (Figure 2B). When we stratified the patients according to the concentration of C4 (less than 25 or 25–50% of normal values) no difference in sPLA_2_ plasma activity between these groups was found (Figure 2C).

**Figure 3:**
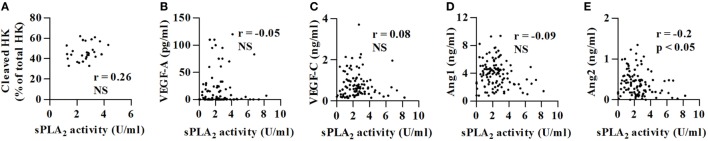
Correlations between sPLA_2_ activity and cleaved HK, VEGF-A, VEGF-C, and Angs concentrations. Correlations between two variables: sPLA_2_ (U/ml) and cleaved HK **(A)**, sPLA_2_ and VEGF-A **(B)**, sPLA_2_ and VEGF-C **(C)**, sPLA_2_ and Ang1 **(D)**, and sPLA_2_ and Ang2 **(E)**, were assessed by Spearman’s correlation analysis and reported as coefficient of correlation (*r*).

We have previously demonstrated that in C1-INH-HAE patients in symptom-free period plasma concentrations of cleaved HK (8) and vascular permeability factors (i.e., VEGFs and Angs) are increased compared with healthy controls (17). Interestingly, sPLA_2_ activity did not correlate with cleaved HK (Figure 3A), VEGF-A (Figure 3B), VEGF-C (Figure 3C), and Ang1 (Figure 3D) concentrations. sPLA_2_ inversely correlated with Ang2 concentrations (*r* = −0.20; *p* < 0.05) (Figure 3E).

### Lack of Association between sPLA_2_ Activity in Plasma and Angioedema Attacks

**Figure 4:**
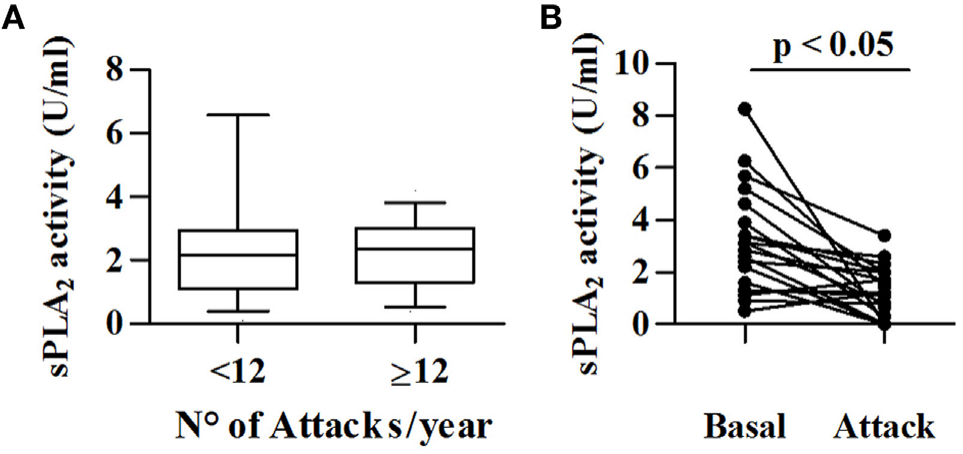
Relationships between sPLA_2_ activity and angioedema attacks. **(A)** sPLA_2_ activity was determined in 69 patients with low frequency (<12/year) and 40 patients with high frequency (>12/year). **(B)** sPLA_2_ activity in plasma collected from 22 patients with C1-INH-HAE patients during symptom-free period (Basal) and during acute attack (Attack).

To investigate a possible role for sPLA_2_s in angioedema attacks, we measured its activity in patients with less (low frequency) or more than 12 attacks (high frequency) in the last 12 months. sPLA_2_ activity was comparable in these two groups (Figure 4A). In addition, we compared sPLA_2_ activity in 22 C1-INH-HAE patients in symptom-free period and during angioedema attacks. We found that the sPLA_2_ activity [attack 1.2 (0.4–2) vs symptom-free period 3.0 (1.4–4.4) U/ml] was reduced in patients examined during attack compared with basal conditions (Figure 4B).

### hGIIA Activity in C1-INH-HAE Plasma increases Endothelial Permeability

**Figure 5:**
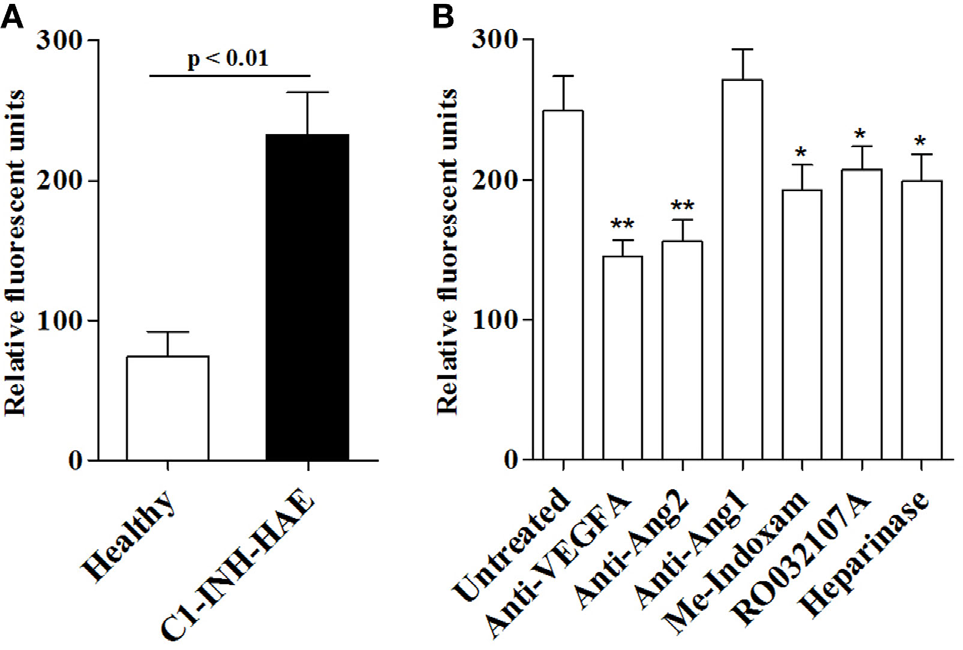
*In vitro* effects of plasma from healthy controls or patients with C1-INH-HAE on vascular permeability. **(A)** Bovine aortic endothelial cells (BAEC) were incubated (18 h, 37°C) with plasma from healthy controls or from symptom-free patients with C1-INH-HAE. The *in vitro* vascular permeability was assessed as indicated in Section “Materials and Methods.” **(B)** Plasma of patients with C1-INH-HAE was incubated (20 min, 37°C) with anti-VEGF-A (1 µg/ml), anti-Ang2 (1 µg/ml), anti-Ang1 (1 µg/ml), Me-Indoxam (100 nM), RO032107A (100 nM), or control medium. BAEC were then pre-incubated (30 min, 37°C) with heparinase (0.4 U/ml) or control medium and stimulated (18 h, 37°C) with plasma of C1-INH-HAE patients alone or with the combination of C1-INH-HAE plasma with inhibitors and then we evaluated vascular permeability. Data are shown in relative fluorescence units. *p Value ≤0.05 and **p value ≤0.01 vs untreated plasma.

Secreted phospholipases A_2_ can modulate endothelial cell mobility and vascular permeability (36). To gain mechanistic insight into the role of sPLA_2_s in C1-INH-HAE, we performed an *in vitro* vascular permeability assay by monitoring the leakage of dextran-FITC through a tight monolayer of BAEC (49). Interestingly, plasma from angioedema patients in symptom-free period increased endothelial permeability compared with control plasma from healthy donors [128 (95–207) vs 52 (8.5–125) RFU] (Figure 5A). To assess whether sPLA_2_ activity in plasma was responsible for this phenomenon, we incubated C1-INH-HAE plasma with blocking antibodies against VEGF-A, Ang1, and Ang2 or Me-Indoxam and RO032107A. Anti-VEGF-A and anti-Ang2 but not anti-Ang1 reduced the effect of C1-INH-HAE plasma without completely abolishing it (i.e., permeability levels were still higher compared with healthy donor plasma) (Figure 5B). Interestingly, Me-Indoxam and RO032107A also reduced vascular permeability induced by C1-INH-HAE plasma (Figure 5B) [untreated: 145 (126–227) vs Me-Indoxam 91 (34–114) RFU]. sPLA_2_s also bind to HSPGs that may mediate some of their biological effects (50–52). To evaluate a possible role for HSPGs, BAEC were treated with heparinase to eliminate surface HSPGs before stimulation with plasma (23). Heparinase treatment of BAEC reduced the endothelial permeability induced by C1-INH-HAE plasma [untreated: 145 (126–227) vs heparinase 117 (92–132) RFU] to a level comparable to Me-Indoxam and RO032107A (Figure 5B).

### hGIIA impairs C1-INH Functional Activity

**Figure 6:**
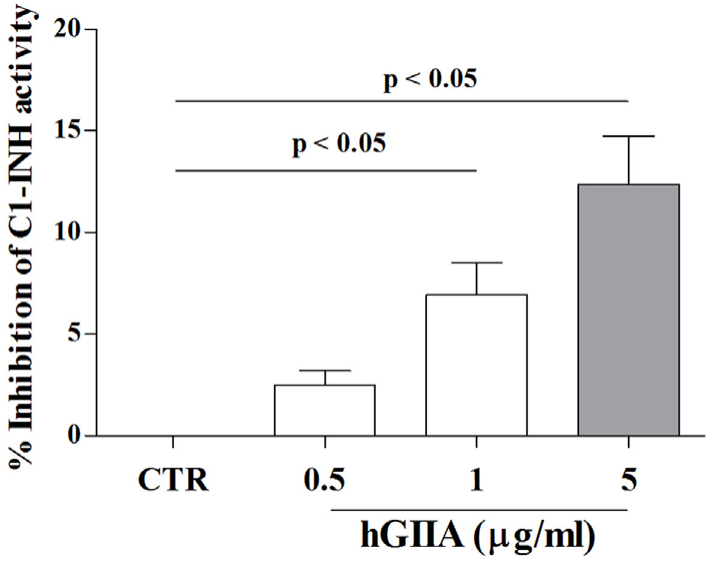
Effect of hGIIA on C1-INH activity. Plasma from normal donors was incubated (2 h, 37°C) with increasing concentrations (0.5–5 µg/ml) of hGIIA or with medium alone and then functional activity of C1-INH was evaluated by colorimetric assay. Data are expressed as percent inhibition of the maximum plasma activity of C1-INH calculated as (*R* − *R*_b_) × 100, where *R* is the C1-INH activity in plasma samples treated with the hGIIA, *R*_b_ is the C1-INH activity in unstimulated samples. Data are the mean ± SD of 15 experiments.

Our results demonstrate that hGIIA activity in C1-INH-HAE plasma increases endothelial leakage *in vitro*, supporting a mechanistic role for sPLA_2_s in modulating vascular permeability *in vivo* (i.e., C1-INH-HAE patients). This effect can be mediated by a direct modulation of endothelial cells through enzymatic activity and/or receptor-mediated mechanisms (e.g., binding to HSPGs). Nevertheless, we hypothesized that hGIIA could also directly affect C1-INH activity. To this aim, we incubated *in vitro* plasma from healthy controls (containing normal C1-INH) with recombinant hGIIA and assessed C1-INH functional activity. Interestingly, hGIIA concentration-dependently reduced C1-INH activity (Figure 6).

## Discussion

In this study, we found that plasma sPLA_2_ enzymatic activity and hGIIA are increased in symptom-free C1-INH-HAE patients compared with healthy controls. sPLA_2_ activity is positively correlated with hGIIA plasma concentrations and inversely correlated with C1-INH activity and protein level. hGIIA in C1-INH-HAE plasma increases endothelial permeability and hGIIA impairs C1-INH functional activity *in vitro*. No correlation was found between plasma sPLA_2_ activity and the severity of angioedema and intriguingly sPLA_2_ activity was decreased during attacks.

The local generation of BK causes angioedema in C1-INH deficiency (10). It is well established that this mediator comes from HK and is cleaved by plasma kallikrein lacking its main physiological inhibitor (11). It is still unclear whether BK generation is sufficient to cause an angioedema attack or if other mechanisms are involved. We have demonstrated that plasma levels of vascular permeability factors (i.e., VEGFs and Angs) are increased in symptom-free C1-INH-HAE patients (17). In this study, we demonstrate that sPLA_2_ activity is increased in plasma of C1-INH-HAE patients and negatively correlates with C1-INH plasma activity. Although multiple sPLA_2_s have been described in mammals, several lines of evidence support a specific role for hGIIA in C1-INH-HAE. First, hGIIA is the most represented sPLA_2_ in human serum and plasma (43–47). Second, hGIIA plasma levels are increased in C1-INH-HAE patients compared with healthy controls. Moreover, hGIIA plasma levels strongly correlates with C1-INH activity. Finally, sPLA_2_ activity in the plasma from both C1-INH-HAE patients and healthy controls is markedly reduced by the hGIIA-specific enzymatic inhibitor RO032107A (39).

Our results suggest a possible involvement of hGIIA in the pathogenesis of C1-INH-HAE. We show that plasma from C1-INH-HAE patients increases endothelial permeability *in vitro* compared with healthy donor plasma. Their effect is partially reverted by the addition of the hGIIA-specific enzymatic inhibitor RO032107A or endothelial cell treatment with heparinase, an enzyme that degrades HSPGs. It is conceivable that hGIIA in C1-INH-HAE plasma binds to HSPGs on endothelial surface and increases endothelial cell permeability in a process that requires its enzymatic activity. These results are in keeping with the evidence that sPLA_2_s can modulate endothelial cell permeability directly or through the release of vasoactive mediators (e.g., PGE_2_, VEGFs, and Angs) (17, 18, 31). Further studies are required to gain more insights into this model and define its relevance to C1-INH-HAE pathogenesis.

We also uncovered another possible mechanism of hGIIA involvement in C1-INH-HAE pathophysiology that is independent of its interaction with endothelial cells. We found that hGIIA impairs C1-INH activity of healthy donor plasma in a concentration-dependent manner. Modulation of C1-INH by other classes of enzymes has already been demonstrated. For example, the proteases elastase and plasmin degrade C1-INH (53, 54). C1-INH also interacts with MBL-associated serine protease 1 (MASP-1), and C1-INH/MASP-1 complexes are reduced in C1-INH-HAE patients (55). Interestingly, both wild-type and catalytically inactive hGIIA bind to factor Xa of the coagulation cascade and inhibit prothrombinase activity (56). Whether hGIIA interacts with C1-INH *in vitro* and *in vivo*, and whether hGIIA enzymatic activity is required for impairing C1-INH activity require further investigation. Our plane is to study a potential interaction between C1-INH and hGIIA, thermodynamically and kinetically, employing single-molecule *in vitro* assays (e.g., surface plasmon resonance and isothermal titration calorimetry). Furthermore, at a later time, it could be possible to detect this interaction on plasma samples.

It has been suggested that a systemic activation process can occur in patients with HAE (4). Accordingly, we have hypothesized that C1-INH deficiency and inflammatory stimuli contribute to generate a variable, ongoing increase in vascular permeability that defines the threshold where localized triggers act for the development of angioedema attack (17). This hypothesis is also supported by previous findings indicating that several proinflammatory mediators such as C reactive protein (57, 58) and pentraxin 3 (57) are elevated in asymptomatic C1-INH-HAE patients and during attacks. Moreover, VEGFs (17) and sPLA_2_ produced by activated immune cells (29, 31, 59–61) are elevated in C1-INH-HAE patients. Together, these findings suggest that low-grade systemic inflammation can occur in these patients.

Comprehensive studies have identified BK as the principal mediator of vascular leakage in C1-INH-HAE-related swelling attacks (10, 62) Circulating levels of BK, markers of endothelial activation, prothrombin fragments, D-dimer (63), cytokines (e.g., TNF-α and IL-8), as well as neutrophil count and neutrophil-derived factors (e.g., elastase, myeloperoxidase, pentraxin 3) (64) are increased during attacks compared with symptom-free periods in C1-INH-HAE patients. By contrast, we found that sPLA_2_ activity is consistently decreased during attacks and does not correlate with canonical biomarkers of angioedema severity (i.e., cleaved HK) (65). Whatever the mechanism(s), sPLA_2_ is the first mediators so far identified which shows opposite behavior during clinical remission (i.e., increase) and angioedema attacks (i.e., reduction). The reasons for this intriguing observation are unclear and command additional *in vitro* and *in vivo* investigations. Furthermore, our preliminary data show that sPLA_2_ activity is not modified by prophylactic treatments (either tranexamic acid or attenuated androgens) compared with untreated patients (unpublished results).

In conclusion, our study provides evidence for a possible role of hGIIA in the pathophysiology of C1-INH-HAE and also gives mechanistic insights into how hGIIA may predispose to the development of angioedema attacks.

## Ethics Statement

The Ethical Committee of the University of Naples Federico II approved that plasma obtained during routine diagnostics could be used for research investigating the physiopathology of hereditary angioedema and written informed consent was obtained from patients in according to the principles expressed in the Declaration of Helsinki. Protocol number 216/16.

## Author Contributions

Substantial contributions to the conception or design of the work; or the acquisition, analysis, or interpretation of data for the work: SL, AF, MB, FB, CS, NV, AP, AZ, HF, MC, and GM. Drafting the work or revising it critically for important intellectual content: SL, AF, MB, FB, CS, NV, AP, MG, GM, GV, FG, AZ, HF, MC, and GL. Final approval of the version to be published; agreement to be accountable for all aspects of the work in ensuring that questions related to the accuracy or integrity of any part of the work are appropriately investigated and resolved: SL, AF, MB, FB, CS, NV, AP, MG, GV, FG, AZ, HF, MC, GL, and GM.

## Conflict of Interest Statement

The authors declare that the research was conducted in the absence of any commercial or financial relationships that could be construed as a potential conflict of interest.

## Abbreviations

Angs, angiopoietins; BK, bradykinin; BAEC, bovine aortic endothelial cells; CRP, C reactive protein; C1-INH-HAE, hereditary angioedema due to C1-inhibitor deficiency; HSPGs, heparan sulfate proteoglycans; HK, high-molecular weight kininogen; RFU, relative fluorescent unit; sPLA_2_s, secreted phospholipases A_2_; hGIIA, human sPLA_2_ group IIA; VEGFs, vascular endothelial growth factors.
