# Supplementary material for: Secreted Phospholipases A2 in Hereditary Angioedema With C1-Inhibitor Deficiency
Source: Front Immunol. 2018 Jul 23;9:1721. doi: 10.3389/fimmu.2018.01721 (PMC6064723; doi:10.3389/fimmu.2018.01721)
Supplement: Supplementary file 1 [file image_1.PDF]

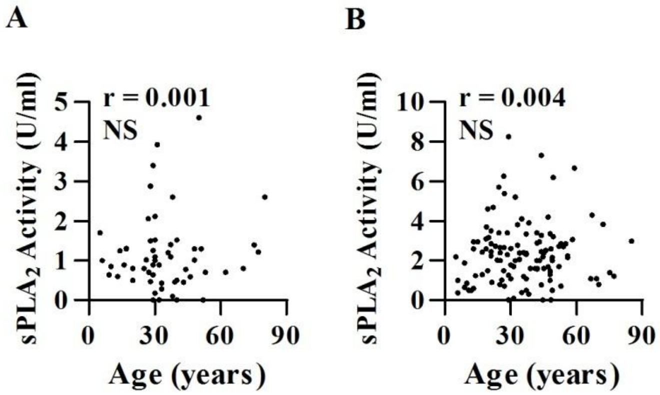

Supplementary Figure 1. Correlations between the age of healthy controls and C1-INH-HAE patients and plasma $sPLA_2$ activity.

$sPLA_2$ in healthy controls (A) and in patients with C1-INH-HAE (B) were determined by immunoenzymatic assay. Correlation between the age (expressed as years) and $sPLA_2$ activity were assessed by linear regression analysis and reported as coefficient of determination (*r*). NS $p > 0.05$.
