# Supplementary material for: Secreted Phospholipases A2 in Hereditary Angioedema With C1-Inhibitor Deficiency
Source: Front Immunol. 2018 Jul 23;9:1721. doi: 10.3389/fimmu.2018.01721 (PMC6064723; doi:10.3389/fimmu.2018.01721)
Supplement: Supplementary file 2 [file table_1.PDF]

Table 1S – $sPLA_2$ plasma activity and gender in healthy and in C1-INH-HAE subjects

	**Healthy**		**C1-INH-HAE**	
	Female	Male	Female	Male
$sPLA_2$ (U/ml)	0.9 (0.4-1.5)	1.3 (0.6-2)	2.2 (1.2-2.9)	2.2 (1.3-2.9)

*Data are expressed as median values (interquartile ranges). Data were analyzed by t test.
